# Hengshun Aromatic Vinegar Improves Glycolipid Metabolism in Type 2 Diabetes Mellitus *via* Regulating PGC-1α/PGC-1β Pathway

**DOI:** 10.3389/fphar.2021.641829

**Published:** 2021-04-26

**Authors:** Guoquan Li, Xuemei Tan, Bao Zhang, Linshu Guan, Yidan Zhang, Lianhong Yin, Meng Gao, Shenghu Zhu, Lina Xu

**Affiliations:** ^1^ School of Food and Biological Engineering, Jiangsu University, Zhenjiang, China; ^2^ Jiangsu Hengshun Vinegar Industry Co., Ltd., Zhenjiang, China; ^3^ College of Pharmacy, Dalian Medical University, Dalian, China

**Keywords:** aromatic vinegar, glucolipid metabolism, PGC-1α, PGC-1β, gluconeogenesis, glycogen synthesis, lipid synthesis

## Abstract

Hengshun aromatic vinegar (HSAV), produced by typical solid-state or liquid-state fermentation techniques, is consumed worldwide as a food condiment. HSAV shows multiple bioactivities, but its activity in type 2 diabetes mellitus (T2DM) and possible mechanisms have not been reported. In this study, the effects of HSAV against T2DM were evaluated in insulin-induced HepG2 cells and high-fat diet (HFD) and streptozotocin (STZ) induced T2DM rats. Then, the mechanisms of HSAV against T2DM were explored by Real-time PCR, Western blot, immunofluorescence assays, siRNA transfection and gene overexpression experiments. Results indicated that HSAV significantly improved glucose consumption and reduced triglycerides (TG) contents in metabolic disordered HepG2 cells. Meanwhile, HSAV obviously alleviated general status, liver and kidney functions of T2DM rats, and decreased hyperglycemia and hyperlipidemia, improved insulin resistance, and reduced lipid accumulation in liver. Mechanism studies indicated that HSAV markedly down-regulated the expression of proliferator-activated receptor γ coactivator-1α (PGC-1α), then regulated peroxisome proliferators-activated receptor α (PPAR-α)/protein kinase B (AKT) signal pathway mediated gluconeogenesis and glycogen synthesis. Meanwhile, HSAV significantly up-regulated proliferator-activated receptor γ coactivator-1β (PGC-1β), and subsequently decreased sterol regulatory element binding protein-1c (SREBP-1c) pathway mediated lipogenesis. In conclusion, HSAV showed potent anti-T2DM activity in ameliorating dysfunction of glycolipid metabolism through regulating PGC-1α/PGC-1β pathway, which has a certain application prospect as an effective diet supplement for T2DM therapy in the future.

## Introduction

Type 2 diabetes mellitus (T2DM) is characterized by chronic hyperglycemia and relative deficiency of insulin, resulting in disorders of lipid, glucose and protein metabolisms (Liang et al., 2020). In 2015, the number of global diabetes cases reached 415 million, which is expected to reach 642 million by 2040 (Ge et al., 2018). Due to the enormous healthcare expenditures, high disability and mortality, T2DM bring huge burden to society and the quality of patients’ life (Sayyid and Fleshner, 2016; Graves et al., 2020; Olm et al., 2020). Therefore, it is an extremely meaningful and urgent work to find effective treatment strategies for T2DM.

The pathogenesis of T2DM is very complicated, and there are many factors affecting its occurrence, including environmental and genetic factors (Ahlqvist et al., 2011). Among them, obesity has been indicated to be closely related to the onset of T2DM, leading to insulin resistance, loss of β-cell mass, adipose tissue dysfunction and chronic low-grade systemic inflammation (Carbone et al., 2019; Kusminski et al., 2020; McCulloch et al., 2020). In particular, the disorder of glycolipid metabolism accompanied by obesity is one of the most critical pathogenesis of T2DM, including aberrant glycogen synthesis, gluconeogenesis, lipogenesis and so on (Sharabi et al., 2015; Jiang et al., 2019). Peroxisome proliferator-activated receptor γ coactivator-1 (PGC-1) family (consisting of PGC-1α, PGC-1β and PRC) mainly exists in brown adipose tissue, heart and liver, as coordinator of energy demand and nutrient supply. PGC-1 family participates in tumorigenesis by balancing the demands of mitochondrial energy production and cell proliferation (Luo et al., 2016). PGC-1α can affect insulin resistance through regulating gluconeogenesis, lipid catabolism, and reactive oxygen species (Besse-Patin et al., 2019; Lv et al., 2019), while PGC-1β can regulate sterol regulatory element binding protein-1c (SREBP-1c) mediated lipogenesis in T2DM (Shuldiner and McLenithan, 2004; Chen et al., 2017). Therefore, regulation of PGC-1α and PGC-1β may ameliorate dysfunction of glucolipid metabolism and relieve T2DM.

There are many kinds of drugs for the treatment of T2DM at present, but some undesirable adverse effects often occur after administrating, including hypoglycemia, fluid retention, osteoporosis and heart failure (Xu et al., 2018a). Hence, it is valuable to find an effective method for the therapy of T2DM with high efficiency and low toxicity. Recently, diet therapy is gradually coming into public view due to its slight side effects and wide sources. Nigella sativa, commonly known as black cumin, is effective in treating cancer, diabetes, dyslipidemia and respiratory diseases (Butt and Sultan, 2010). Garlic has been proved to be beneficial to atherosclerosis, hypertension and diabetes, and helps reduce the risk of myocardial infarction and ischemic stroke (Zhu et al., 2018). Consumption of refined grain has been reported to be linked to insulin resistance and metabolic syndrome in urban south Indian population (Radhika et al., 2009). These findings suggest that dietary therapy will open new avenues to prevent various diseases.

Vinegar contains a variety of nutrients, and has been used as acidic condiment more than three thousand years around the world (Budak et al., 2014). The main component of vinegar is acetic acid, which increases glycogen supplementation of liver and skeletal muscle by gluconeogenesis and inhibition of glycolysis, and has a fatigue recovery effect on rats (Fushimi et al., 2001). Cider vinegar shows antidiabetic effects and can decrease cholesterol levels by inhibiting the oxidation of low density lipoproteins (LDLs) (Liljeberg and Björck, 1998; Shishehbor et al., 2008). Furthermore, vinegar intake prevented metabolic syndrome by reducing body weight, body fat mass, and serum triglyceride levels in obese Japanese subjects (Kondo et al., 2009). Hengshun aromatic vinegar (HSAV), a representative product of traditional solid-state fermented vinegar in China, is brewed with rice or glutinous rice, rice husk and rice bran. The production process of HSAV mainly includes three main steps: alcohol fermentation, acetic acid fermentation and aging (Yu et al., 2020). HSAV contains a large number of antioxidant compounds, including polyphenols, flavonoids, and melanosine, which play a key role in the prevention of diseases and beneficial to human health (Zhao et al., 2018). Our previous study found that HSAV showed potent effect against non-alcoholic fatty liver disease (NAFLD) through regulating lipid metabolism and ameliorating inflammation (Zhu et al., 2020). The previous findings gave a hint that HSAV may have therapeutic effect on T2DM, which can be further investigated. Thus, effects of HSAV on T2DM were evaluated in insulin induced HepG2 cells and streptozotocin combined with high-fat diet induced rats, and the molecular mechanism was further elucidated in this study.

## Methods

### Chemicals and Materials

HSAV was produced by Jiangsu Hengshun Vinegar Industry Co., Ltd. (Zhenjiang, China), and was diluted to the suitable concentrations with purified water. Glibenclamide was purchased from MedChemExpress (Shanghai, China) and was dissolved in the normal saline containing 10% DMSO, 40% PEG300 and 5% Tween-80 for rat administration.

### Cell Culture

Human hepatocellular carcinoma HepG2 cells were purchased from Shanghai Institute of Biochemistry and Cell Biology (Shanghai, China), and cultured in Dulbecco’s Modified Eagle’s medium (DMEM) supplemented with 10% fetal bovine serum (FBS, California, United States) in humidified air containing 5% CO_2_ and 95% O_2_ at 37°C.

### Cytotoxicity of HSAV and Model Development in HepG2 Cells

To select the safe concentrations of HSAV, the cytotoxicity of HSAV was evaluated in HepG2 cells. Cells were incubated in 96-well at a density of 5 × 10^4^ cells/well overnight, and different concentrations of HSAV (0.125, 0.25, 0.5, 1, 1.5, 2, 3, and 4%) were added and incubated for 12, 24, and 36 h, respectively. The cell viabilities were evaluated by CCK-8 assay (n = 6). To select a suitable condition for model development, HepG2 cells were administrated with different concentrations (1 × 10^–8^, 1 × 10^–7^, 1 × 10^–6^ and 1 × 10^–5^ mol/L) of insulin (Solarbio, Beijing, China) for 12, 24 and 36 h, and then the cell viabilities were detected (n = 6).

### HSAV Intervention in Insulin-Induced HepG2 Cells

HepG2 cells were incubated in 96-well at a density of 5 × 10^4^ cells/well overnight. The culture medium was changed to DMEM containing 0.5, 1.0 or 1.5% HSAV (final concentration), respectively. After 24 h incubation, culture medium of cells was changed to DMEM containing 10^–6^ mol/L insulin. After another 24 h incubation, cell viability, glucose consumption and TG content were detected according to the manufacturer’s instructions (n = 6).

### Animals and Experimental Design

Male Sprague Dawley (SD) rats (weighing 180–220 g) were purchased from Liaoning Changsheng Biotechnology Co., Ltd. (SCXK (Liao) 2015–0,001, Benxi, China). Rats were housed with a 12:12 h of light/dark cycle at an ambient temperature of 22–25°C for 7 days. Animal experiments were approved by the Animal Care and Use Committee of Dalian Medical University (SYXK (Liao) 2013–0,006), and all experimental procedures were conducted in accordance with the People's Republic of China regulations on the use and care of laboratory animals. In this study, T2DM model of rats was established by high-fat diet (HFD, consisting of 10% fat, 20% glucose, 2.5% cholesterol, 1% cholate, 1% egg, 30% bean sprout and 35.5% chow diet) and a single low-dose intraperitoneal injection of streptozotocin (STZ, 40 mg/kg). Initially, rats were randomly divided into two groups: HFD group and normal diet group. Rats in HFD group were fed HFD for 4 weeks, and then given an intraperitoneal injection of a single dose of STZ (40 mg/kg) (dissolved in citrate buffer at pH 4.5, Saiguo Biotechnology, Guangzhou, China) to induce T2DM (An et al., 2018). Rats in normal diet group were fed chow diet for the same period, and given an intraperitoneal injection of citrate buffer. Three days after STZ injection, the glucose levels of blood collected from the tail vein were measured. Rats with random blood glucose >16.6 mmol/L (Shao Q. et al., 2019) were considered as T2DM rats. The T2DM rats were randomly divided into five groups: model group (normal saline), HSAV-treated groups (0.625 ml/kg, 0.833 ml/kg and 1.250 ml/kg HSAV), and positive drug group (10 mg/kg glibenclamide). Rats in normal diet group were randomly divided into control group (normal saline) and aromatic vinegar control group (1.25 ml/kg HSAV). There were 10 rats in each group. Then, corresponding drugs were intragastrically administrated to rats once a day (10 ml/kg) for consecutive 4 weeks. Finally, rats were sacrificed after a 12 h fast, and liver, kidney tissues and serum were collected for the following tests.

### Body Weight, Food Intake and Water Intake

The body weight of rats was recorded weekly during the experiment. The food intake and water intake were recorded in the last week before the rats were sacrificed.

### Examinations of Liver and Renal Index, and Biochemical Indicators

The weights of liver and renal were weighed and the liver and renal indexes were calculated, respectively. The levels of alanine aminotransferase (ALT), aspartate aminotransferase (AST), creatinine (CRE), urea nitrogen (BUN), triglyceride (TG), total cholesterol (TC), free fatty acid (FFA), low density lipoprotein (LDL), and high-density lipoprotein (HDL) were measured by kits according to the manufacturer’s instructions (Jiancheng Institute of Biotechnology, Nanjing, China).

### Glucose Metabolism Test

The levels of liver glycogen (Solarbio Science and Technology, Beijing, China), serum insulin (INS) (CUSABIO, Wuhan, China) and glycosylated hemoglobin (GHb, Mei biao Biological Technology, Jiangsu, China) were measured by kits according to protocols. In the last week of administration, oral glucose tolerance test (OGTT) was performed. Rats were fasted for 6 h and orally administrated with 2 g/kg glucose. At 0, 30, 60, 90 and 120 min after glucose administrated, blood glucose level was measured (Xu et al., 2018b). Then, the area under the blood glucose-time curve (AUC) in OGTT was calculated *via* trapezoidal method using GraphPad Prism 5.0 software (Paragraph Software, CA, United States) (Ghane et al., 2019). In addition, homeostasis model assessment for insulin resistance index (HOMA-IR) and insulin sensitivity index (ISI) were calculated to assess the degrees of insulin resistance and sensitivity (Zhang et al., 2012; Jiao et al., 2017). The formulas are as follows:

HOMA-IR=FBG [mmol/L] × FINS (fasting insulin) [nIU/L]/22.5

ISI=1/(FBG × FINS)

### Histological Analyses

Liver and kidney tissues were buried in 4% polyformaldehyde solution for 24 h. Then, formalin-fixed liver and kidney tissues were embedded in paraffin. Then, liver and kidney sections with 5 μm thick were stained with hematoxylin-eosin (H&E). Furthermore, the liver sections were also stained with periodic acid-schiff stain (PAS) to observe hepatic glycogen. The frozen liver tissue slices were stained with Oil Red O to evaluate lipid droplet accumulation. Images were acquired by a light Nikon Eclipse TE2000-U microscope (NIKON, Japan) with 200× magnification.

### Immunofluorescence Assay

The paraffin sections of liver were dewaxed and incubated with anti-PGC-1α antibodies in a humidified box at 4°C overnight. On the next day, the sections were incubated with alexa fluorescein-labeled secondary antibody out of light for 1 h at 37°C. Then, the sections were stained with 4′,6-diamidino-2-phenylindole (DAPI, 1.0 μg/ml) for 10 min. After washed with PBS, the images were obtained by a TE 2000U fluorescence microscopy (Nikon) with 200× magnification.

### Western Blotting Assay

Total proteins of liver tissues and HepG2 cells were extracted by cold lysis buffer (Solarbio Science and Technology, Beijing, China) containing 1 mmol/L phenylmethyl sulfonyl fluoride. Then bicinchonininc acid (BCA) protein assay kit (TransGen Biotechnology, Beijing, China) was used to detect the protein concentration. Proteins were dispersed through SDS-PAGE (8–12%) and then transferred to PVDF membranes (Millipore, MA, United States). After blocking nonspecific binding sites with 5% dried skim milk, the membranes were individually incubated with the primary antibodies for 4°C overnight. Then, specific antibody binding was detected by horseradish peroxidase-conjugated secondary antibody for 2 h incubation at room temperature. Protein expression was detected by enhanced chemiluminescence (ECL) method with a Bio-Spectrum Gel Imaging System (UVP, CA, United States). The relative expression of target proteins was normalized to that of β-actin.

### Real-Time PCR Assay

Total RNA were extracted using TransZolTM, and reverse-transcribed into cDNA according to the instructions of the Prime Script^®^ RT kit (TransGen Biotech, Beijing, China). Real-time PCR experiment was carried out on CFX96 PCR system (Bio-Rad Laboratories, United States). β-actin was used to normalize mRNA levels, and the relative mRNA levels were calculated using the 2^−△△Ct^ method.

### Overexpression of PGC-1α in HepG2 Cells

HepG2 cells were incubated in 6-well plates for 24 h and then transfected with PGC-1α (Genepharma, Shanghai, China)or empty plasmid (negative control, NC) using lipofectamine 2000 (Thermo Fisher Scientific, Shanghai, China) for 6 h. After discarding the medium, the cells were added fresh medium containing 10% fetal bovine serum and incubate for 24 h. Then, the cells were pretreated with HSAV for 24 h and exposed to insulin for an additional 24 h. The cells were then collected for the subsequent experiments.

### Silencing of PGC-1β With siRNA Transfection in HepG2 Cells

HepG2 cells were incubated in 6-well plates for 24 h and then transfected with PGC-1β siRNA (Genepharma) or empty plasmid (NC) using lipofectamine2000 for 6 h. After discarding the medium, the cells were added fresh medium containing 10% fetal bovine serum and incubate for 24 h. Then, the cells were pretreated with HSAV for 24 h and exposed to insulin for an additional 24 h. The cells were collected for subsequent experiments.

### Statistical Analysis

Data are expressed as mean ± standard deviation (SD). Statistical analysis was performed using GraphPad Prism 5.0 software. Significant differences among multiple groups were analyzed by one-way ANOVA followed by Newman–Keuls test. An unpaired t-test was carried out in the comparison two different groups. The results were considered to be statistically significant at p < 0.05. The data and statistical analysis were in accordance with standard recommendations for pharmacological experiment design and analysis.

## Results

### Effect of HSAV on Insulin-Induced HepG2 Cells

**FIGURE 1:**
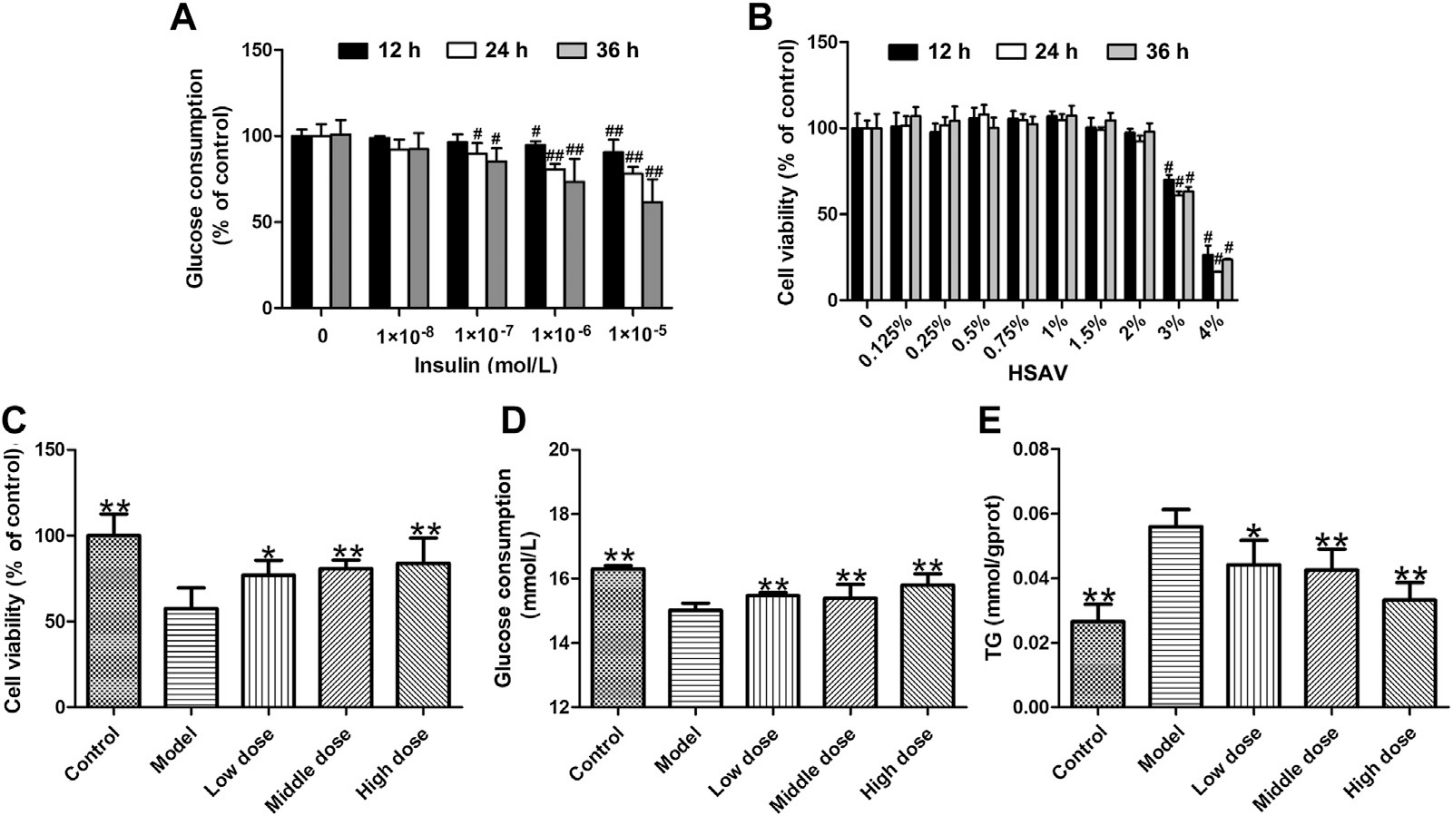
Effects of Hengshun aromatic vinegar (HSAV) on insulin-induced HepG2 cells. **(A)** Effect of insulin on glucose consumption of HepG2 cells; **(B)** Cytotoxicity effect of HSAV on HepG2 cells; Effect of HSAV on cell viability **(C)**, glucose consumption **(D)** and TG content **(E)** in insulin-induced HepG2 cells. Data are presented as the mean ± SD (n = 6). ^#^ *p* < 0.05 and ^##^ *p* < 0.01 compared with control group; *p < 0.05 and **p < 0.01 compared with model group.

In this study, insulin-induced HepG2 cells were used to explore the effect of HSAV on metabolic disorder *in vitro*. In order to select the suitable concentration for modeling, HepG2 cells were treated with different concentrations of insulin, and the results showed that the glucose consumption of cells was markedly decreased when the cells were incubated with 10^–6^ mol/L insulin for 24 h (*p* < 0.01) (Figure 1A). Then, treatment with 10^–6^ mol/L of insulin for 24 h was selected to establish metabolic disorder model in HepG2 cells. The cytotoxicity of HSAV was investigated in HepG2 cells, and the results indicated that the cell viability was significantly affected when the concentration of HSAV was higher than 2% (*p* < 0.05, Figure 1B). Culture medium containing 0.5, 1.0 and 1.5% HSAV were chosen in the following *in vitro* experiments. Compared with model group, HSAV pretreatment markedly improved the cell viabilities of HepG2 cells treated by insulin (*p* < 0.05, Figure 1C), and obviously increased glucose consumption (*p* < 0.01, Figure 1D). Furthermore, HSAV markedly decreased TG content in insulin-induced HepG2 cells compared with model group (*p* < 0.01, Figure 1E).

### Effects of HSAV on Body Weight, Food and Water Intake of T2DM Rats

**FIGURE 2:**
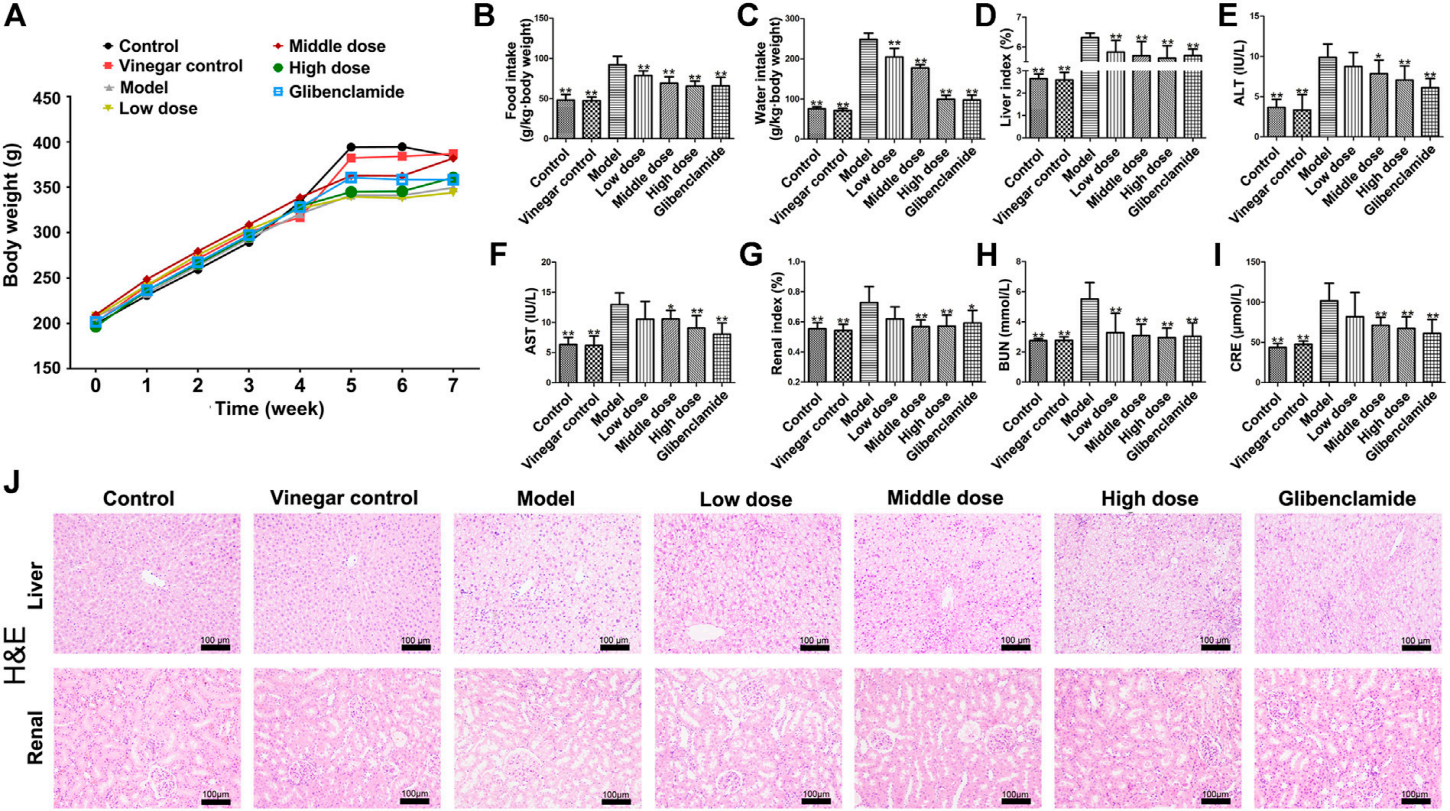
Effects of HSAV on general status and liver functions of T2DM rats. **(A)** Body weight; **(B)** food intake; **(C)** water intake; **(D)** liver index; **(E)** ALT; **(F)** AST; **(G)** renal index; **(H)** BUN; **(I)** CRE; **(J)** H&E staining of liver and renal tissue. Data are presented as the mean ± SD (n = 10). *p < 0.05 and **p < 0.01 compared with model group.

The body weights of rats in each group are as shown in Figure 2A. In the first 4 weeks, there was no significant difference in the body weight of rats among different groups. Due to the induction of T2DM, the body weights of rats in model group were lower than those of control group. However, HSAV improved the body weights of rats with T2DM in comparison with model group. Consistently, the food intake and water intake of rats in model group were markedly increased (*p* < 0.01) (Figure 2B,C), but they were significantly reduced by HSAV or glibenclamide administration compared with model group (*p* < 0.01). These results demonstrated that HSAV ameliorated general state of rats with T2DM.

### Effect of HSAV on the Liver Injury of T2DM Rats

HSAV significantly reduced the liver index compared with model group (*p* < 0.01, Figure 2D ). The serum levels of ALT and AST of rats in model group were obviously higher than those of control group (*p* < 0.01). Administration of HSAV (middle and high dose) or glibenclamide declined the levels of ALT and AST compared with model group (*p* < 0.05, Figure 2E,F). H&E staining showed that the liver exhibited hepatic stestosis and swelling in model groups, but HSAV ameliorated those histopathological changes (Figure 2J).

### Effect of HSAV on the Renal Injury in T2DM Rats

As shown in Figure 2G, HSAV obviously decreased renal index compared with model group (*p* < 0.05). The serum BUN level of rats in HSAV or glibenclamide group was lower than that of model group (*p* < 0.01, Figure 2H). Compare to model group, high dose of HSAV and glibenclamide decreased CRE level in serum (*p* < 0.01, Figure 2I). H&E staining showed that the kidney tissue became lightly stained and glomerular hypertrophy, while HSAV significantly alleviated such lesions (Figure 2J). Collectively, these data showed HSAV can alleviate renal injury in T2DM rats.

### Effect of HSAV on Random Blood Glucose (RBG), INS, GHb and Liver Glycogen Content in T2DM Rats

**FIGURE 3:**
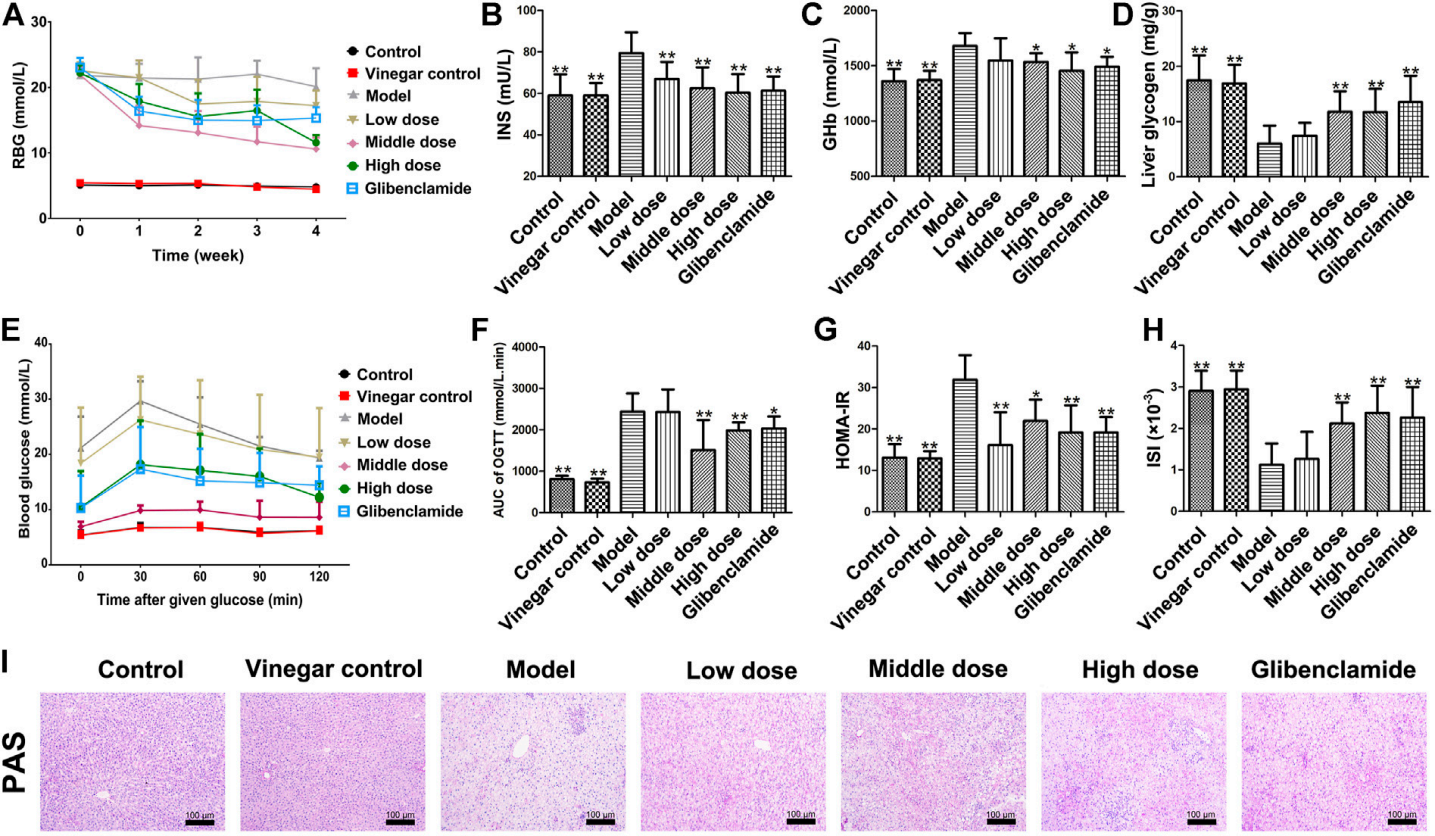
Effect of HSAV on glycometabolism of T2DM rats. **(A)** Random blood glucose (RBG) (Weekly after administration); **(B)** insulin (INS) level in serum; **(C)** GHb level in serum; **(D)** liver glycogen content; **(E)** OGTT; **(F)** AUC of OGTT; **(G)** HOMA-IR; **(H)** ISI; **(I)** PAS staining of liver tissue. Data are presented as the mean ± SD (n = 10). *p < 0.05 and **p < 0.01 compared with model group.

Figure 3A show the variation of RBG in each group with the extension of administration time. After a week of administration, RBG level of rats treated with HSAV was lower than that of model group. The hypoglycemic effect of HSAV was the most obvious at the 4th week. Meanwhile, compared with control group, INS and GHb levels were significantly increased in model group, while HSAV and glibenclamide obviously decreased these indicators (*p* < 0.05, Figure 3B,C). Furthermore, the content of liver glycogen in T2DM rats was markedly lower than control group (*p* < 0.01), while administration of HSAV or glibenclamide increased liver glycogen content (*p* < 0.01, Figure 3D). The result was further confirmed by PAS staining (Figure 3I).

### Effect of HSAV on OGTT in T2DM Rats

In OGTT, blood glucose level in model group was much higher than control group after given glucose, while it was lower in HSAV or glibenclamide group than model group (Figure 3E). Accordingly, the value of OGTT-AUC in model group was much higher than that of control group (*p* < 0.01), and HSAV or glibenclamide reduced OGTT-AUC as shown in Figure 3F (*p* < 0.05). Furthermore, we also found that rats in middle dose of HSAV showed the best glucose tolerance in OGTT.

### Effect of HSAV on HOMA-IR and ISI in T2DM Rats

As shown in Figure 3G, the HOMA-IR value of model group was significantly higher than control group (*p* < 0.01). After 4 weeks of treatment, HSAV and glibenclamide markedly reduced HOMA-IR value compared with model group (*p* < 0.05). Moreover, ISI remarkably declined in model group compared with control group (*p* < 0.01), but it was raised by HSAV or glibenclamide treatment (*p* < 0.01, Figure 3H).

### Effect of HSAV on Lipid Metabolism Indexes in T2DM Rats

**FIGURE 4:**
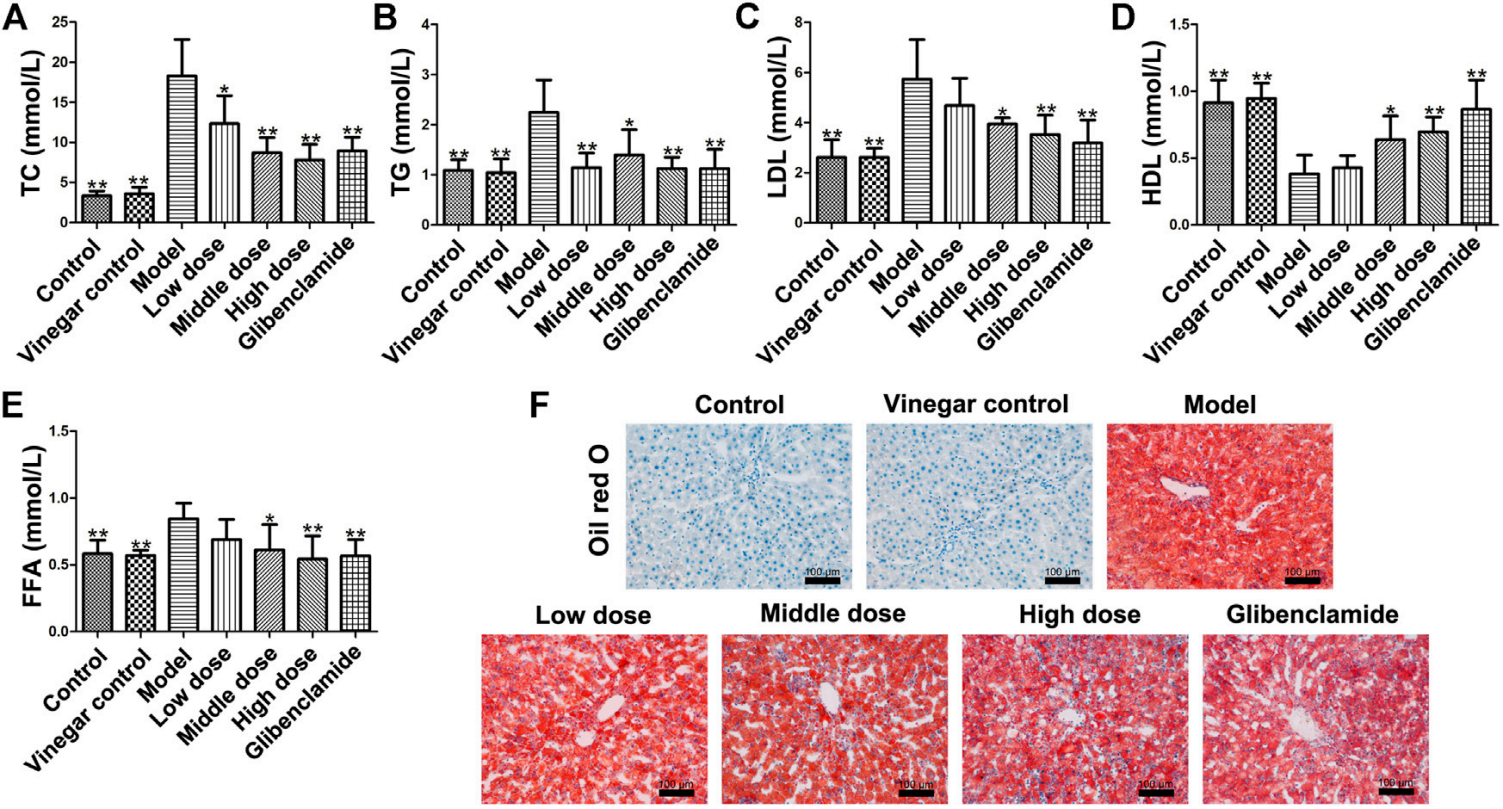
Effect of HSAV on lipid metabolism of T2DM rats. **(A)** TC; **(B)** TG; **(C)** LDL-C; **(D)** HDL-C; **(E)** FFA; **(F)** Oil Red O staining of liver tissue. Data are presented as the mean ± SD (n = 10). *p < 0.05 and **p < 0.01 compared with model group.

The levels of TC, TG, LDL and FFA were obviously ascended in model group (*p* < 0.01), but administration of HSAV or glibenclamide markedly decreased those indicators compared with model group (Figure 4A–C,E). At the same time, middle and high dose of HSAV markedly increased HDL level (*p* < 0.05, Figure 4D). Oil red O staining also showed that HSAV and glibenclamide can reduce the accumulation of lipid droplets in liver tissue (shown in Figure 4F).

### Effects of HSAV on the Expressions of PGC-1α and PGC-1β

**FIGURE 5:**
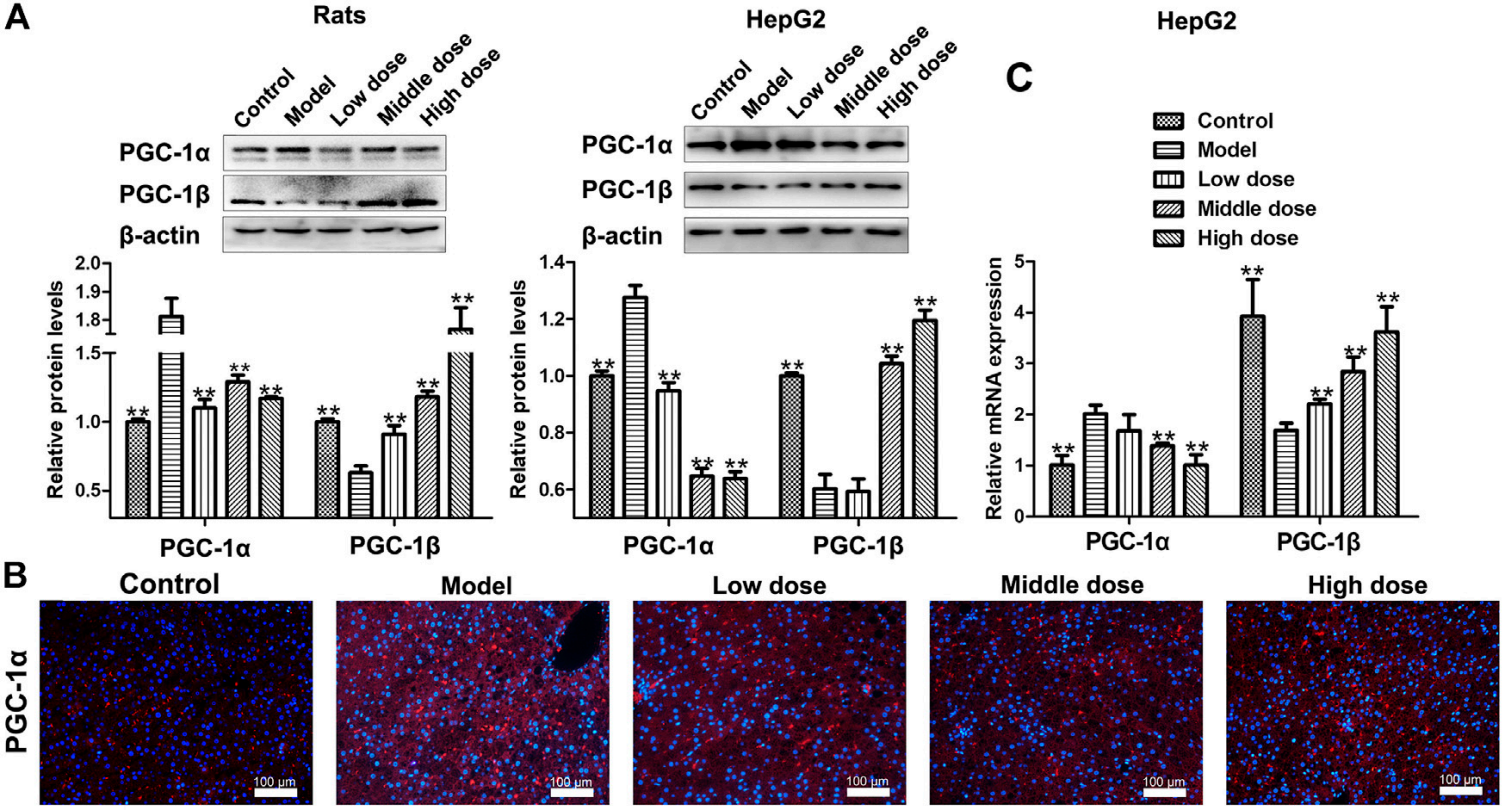
Effect of HSAV on the expression of PGC-1α and PGC-1β. **(A)** The expression levels of PGC-1α and PGC-1β detected by Western blot. **(B)** The expression of PGC-1α in the liver of rats analyzed by immunofluorescence. **(C)** The mRNA levels of PGC-1α and PGC-1β in HepG2 cells analyzed by Real-time PCR. Data are presented as the mean ± SD (n = 3). *p < 0.05 and **p < 0.01 compared with model group.

The expression of PGC-1α was up-regulated in model groups, while it was significantly down-regulated by HSAV treatment in Western blot assay (*p* < 0.01, Figure 5A). The same trend of PGC-1α expression in each group was also confirmed by immunofluorescence (Figure 5B). Nevertheless, the expression of PGC-1β in model group remarked declined compared with control group (*p* < 0.01), while HSAV obviously reversed the abnormal expression of PGC-1β (*p* < 0.01, Figure 5A). Real-time PCR results showed that HSAV also significantly decreased the mRNA level of PGC-1α (*p* < 0.01) and increased the mRNA level of PGC-1β (*p* < 0.01) comepared with model group *in vitro* (Figure 5C).

### Effects of HSAV on the Expressions of PPAR-α and *p*-AKT/AKT

**FIGURE 6:**
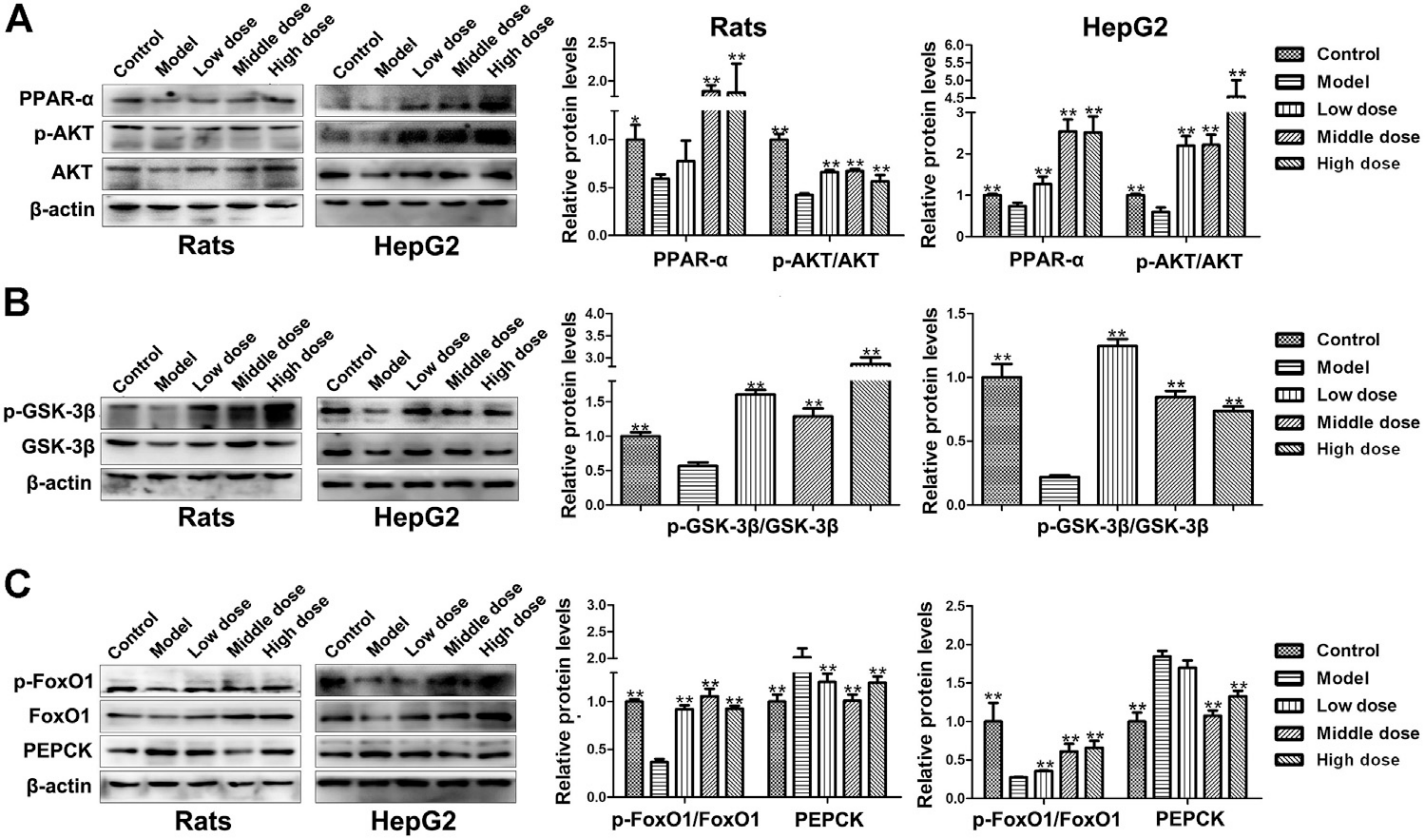
Effect of HSAV on the expression of downstream proteins of PGC-1α by Western blot. **(A)** The expression levels of PPAR-α, AKT and *p*-AKT; **(B)** the expression levels of GSK-3β and *p*-GSK-3β; **(C)** the expression levels of FoxO1, *p*-FoxO1 and PEPCK. Data are presented as the mean ± SD (n = 3). *p < 0.05 and **p < 0.01 compared with model group.

The expression level of peroxisome proliferators-activated receptor α (PPAR-α) as decreased in model group compared with control group (*p* < 0.01), but it was upregulated by HSAV treatment (*p* < 0.01, Figure 6A). Likewise, the phosphorylation level of protein kinase B (*p*-AKT) went down in model group, which was up-regulated in HSAV group in comparison with model group (*p* < 0.01, Figure 6A).

### Effects of HSAV on the Expressions of Proteins Associated With Glucose Metabolism

As shown in Figure 6B, compared with control group, the phosphorylation level of glycogen synthase kinase-3β (*p*-GSK-3β) significantly declined in model group (*p* < 0.01), while HSAV reversed its expression. Meanwhile, the phosphorylation levels of forkhead transcription factors 1 (*p*-FoxO1) was declined (*p* < 0.01), and the expression levels of phosphoenolpyruvate carboxykinase (PEPCK) was accordingly increased in model group (*p* < 0.01, Figure 6C). HSAV markedly up-regulated the expression of *p*-FoxO1 (*p* < 0.01) and down-regulated PEPCK expression compared with model group (*p* < 0.01, Figure 6C).

### Effects of HSAV on the Expressions of Proteins and Genes Related to Lipid Metabolism

**FIGURE 7:**
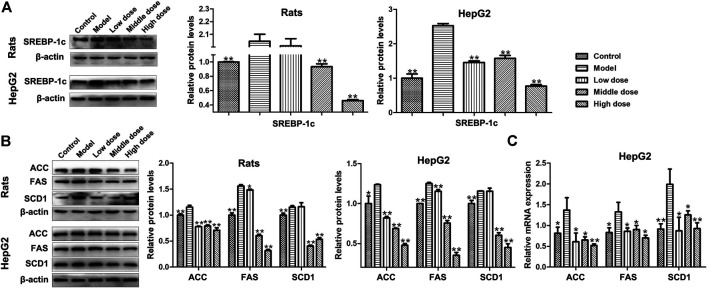
Effect of HSAV on the expressions of downstream proteins related to PGC-1β mediated lipid metabolism pathway. **(A)** The expression level of SREBP-1c by Western blot; **(B)** the expression levels of ACC, FAS, and SCD1 by Western blot; **(C)** the mRNA levels of ACC, FAS, and SCD1 in HepG2 cells by Real-time PCR. Data are presented as the mean ± SD (n = 3). *p < 0.05 and **p < 0.01 compared with model group.

Due to the reduction of PGC-1β, the expression level of SREBP-1c was increased in model groups, but HSAV decreased the expression level of SREBP-1c compared with model group (*p* < 0.01, Figure 7A). Meanwhile, the expression levels of downstream proteins of SREBP-1c were all decreased by HSAV treatment (*p* < 0.05, Figure 7B), containing fatty acid synthase (FAS), acetyl-coenzyme a carboxylase (ACC) and stearoylcoa desaturase-1 (SCD1). HSAV also reduced the mRNA levels of FAS, ACC and SCD1 in HepG2 cells compared with model group (*p* < 0.05, Figure 7C).

### Effects of HSAV on PGC-1α Pathway in HepG2 Cells With PGC-1α Overexpression

**FIGURE 8:**
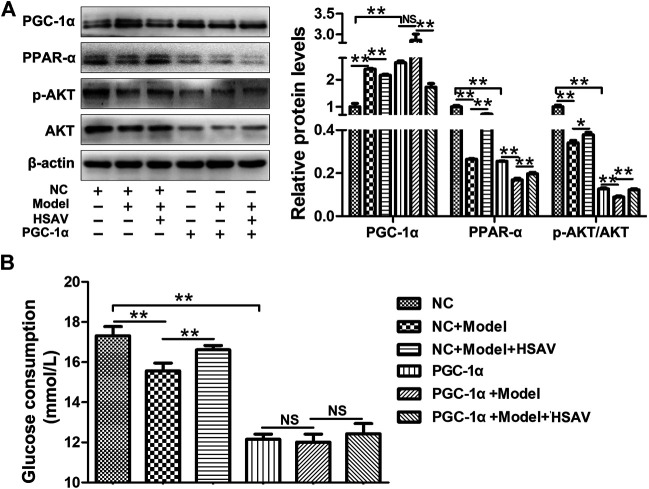
Effects of HSAV on PGC-1α pathway in HepG2 cells with PGC-1α overexpression. **(A)** The expression levels of PGC-1α, PPAR-α, AKT and *p*-AKT (n = 3); **(B)** glucose consumption (n = 6). Data are presented as the mean ± SD. *p < 0.05, **p < 0.01, NS, no significance.

After overexpression of PGC-1α in HepG2 cells, the expression level of PGC-1α was markedly up-regulated compared with NC group, and the expression levels of PPAR-α and *p*-AKT/AKT were markedly down-reguglated in PGC-1α overexpression group (*p* < 0.01, Figure 8A). HSAV reversed the changes of those protein expressions induced by PGC-1α overexpression (Figure 8A). In addition, glucose consumption of HepG2 cells was significantly lower than NC group after PGC-1α overexpression (*p* < 0.01), and there was no significant change after HSAV treatment (Figure 8B).

### Effects of HSAV on PGC-1β Pathway in HepG2 Cells Transfected With PGC-1β siRNA

**FIGURE 9:**
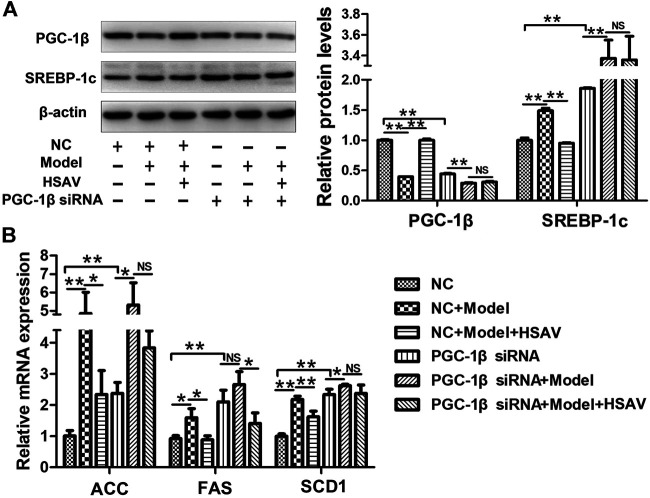
Effects of HSAV on PGC-1β pathway in HepG2 cells transfected with PGC-1β siRNA. **(A)** The expression levels of PGC-1β and SREBP-1c; **(B)** the mRNA levels of ACC, FAS and SCD1. Data are presented as the mean ± SD (n = 3). *p < 0.05, **p < 0.01, NS, no significance.

HepG2 cells were transfected with PGC-1β siRNA to further determine the effect of HSAV on PGC-1β pathway. As shown in Figure 9A, compared with NC group, the expression level of PGC-1β was obviously decreased (*p* < 0.01) and the expression level of SREBP-1c was obviously increased in HepG2 cells transfected with PGC-1β siRNA (*p* < 0.01). As a consequence, the mRNA levels of ACC, SCD1 and FAS were significantly upregulated by PGC-1β siRNA transfection compared with NC group (*p* < 0.01, Figure 9B). Furthermore, PGC-1β siRNA transfection blocked the effects of HSAV on the protein expressions of PGC-1β and SREBP-1c, and the mRNA levels of ACC, SCD1 and FAS (Figure 9A,B).

## Discussion

T2DM is a chronic disorder, which can cause a variety of complications including cardiovascular disease, renal and urogenital disorder (Zheng et al., 2018; Deerochanawong et al., 2019). As we all know, the clinical manifestations of T2DM mainly include polydipsia, polyphagia, polyuria and emaciation. It has been documented that glycolipid metabolism, inflammation and oxidative stress are involved in the pathogenesis of T2DM (Rehman et al., 2020). Among them, dysfunction of glycolipid metabolism is getting more and more researchers’ attention. New treatment strategies that can impove glycolipid metabolism are increasing needed due to the ineffective control of T2DM.

HSAV, one of the most famous vinegar in China, contains various physicochemical and bioactive compositions including organic acids, amino acids and phenolic compounds (Zhang et al., 2019). It has been indicated that polyphenol-rich extract of aromatic vinegar can increase glucose uptake and glucose consumption in high glucose-induced insulin resistant HepG2 cells (Xia et al., 2021). Meanwhile, a bioactive component of aromatic vinegar named ligustrazine has been used as clinical medication for cerebral thrombosis, coronary heart disease and stenocardia recently (Xu et al., 2011; Zou et al., 2018). Therefore, to deeply understand the medicinal value, we explored whether HSAV could play a role in T2DM therapy and elucidated the potential molecular mechanisms.

The pharmacological effects of HSAV were evaluated both *in vitro* and *in vivo*. In insulin-induced HepG2 cells, HSAV significantly increased the glucose comsumption, and reduced TG content. In T2DM rats, HSAV obviously improved the general states, liver and kidney function of rats, and ameliorated the histopathological changes in liver and renal tissues. Moreover, HSAV significantly decreased levels of blood glucose, INS and GHb, increased liver glycogen content, and improved insulin sensitivity and glucose tolerance of T2DM rats. In lipid metabolism, HSAV remarkably reduced lipid level in serum and lipid accumulation in the liver. Strikingly, HSAV evidently alleviated dysfunctions of glucose and lipid metabolism, and showed an excellent anti-T2DM effect.

PGC-1α is a transcriptional coactivator expressed as multiple, alternatively spliced variants transcribed from different promoters. PGC-1α is not directly bound to DNA but is recruited to the template *via* interacting with diverse transcription factors involved in cellular energy metabolism. By regulating those transcription factors, PGC-1α acts as a molecular switch in multiple cellular processes, including mitochondrial biogenesis and respiration, gluconeogenesis and glucose transport, glycogenolysis (Wu et al., 2016; Lee et al., 2019; Léveillé et al., 2020). Studies have shown that dysregulation of PGC-1α can cause abnormal gluconeogenesis and glycogen synthesis that have an important link to the occurrence of T2DM (Ruan et al., 2012; Chan and Arany, 2014). Reversing abnormal PGC-1α expression can change the aberrant gluconeogenesis and glycogen synthesis in T2DM (Xu X. et al., 2018; Gu et al., 2019). Hence, PGC-1α has been considered as a promising target for anti-diabetes therapy. This study uncovered that HSAV significantly decreased the abnormal upregulation of PGC-1α level caused by T2DM model *in vitro* and *in vivo*.

PGC-1α binds numerous transcription factors involved in regulating lipid and glucose metabolism after activation. PPAR-α is one of such transcription factor that can partially mediate the downstream signaling effects (Khan et al., 2015; Yuan et al., 2019). PPAR-α can improve dysfunction of glucose metabolism in T2DM by increasing insulin sensitivity, reducing hyperglycaemia and hyperinsulinaemia (Hong et al., 2018; Zhang et al., 2018; Xu et al., 2006), and alleviate insulin resistance to decrease the incidence of diabetes (Feng et al., 2019). In addition, studies have demonstrated that PPAR can activate AKT expression, resulting in increased insulin sensitivity and improvement of glucose metabolism in diabetes mellitus (Shao Y. et al., 2019; Zhao et al., 2020). The inhibition of AKT phosphorylation can subsequently suppress GSK-3β phosphorylation and affect the activity of glycogen synthase (Yang et al., 2017; Zhang et al., 2017). Similarly, FoxO1 is another downstream of AKT, which mediates glucose production in the liver through regulating gluconeogenesis gene, such as PEPCK (Puigserver et al., 2003). In this study, we found that HSAV significantly increased the expression of PPAR-α and then consequently inhibited the phosphorylation level of AKT. Afterward, the levels of *p*-GSK-3β and *p*-FoxO1 were up-regulated, and PEPCK was down-regulated by HSAV, which elucidated that HSAV remodeled glycogen synthesis and gluconeogenesis in T2DM by regulating PGC-1α signal pathway.

PGC-1β is involved in the development of various diseases, including liver cancer, diabetic cardiomyopathy, and hyperlipidemia (Chen et al., 2017; Li et al., 2019; Yin et al., 2019). PGC-1β can reduce fat accumulation in the liver and greatly increase circulating TC and cholesterol in VLDL particle. PGC-1β is also a key regulator in lipid metabolism in the development of diabetes (Shuldiner and McLenithan, 2004; Ko et al., 2019). In the transcriptional regulation of lipogenesis, PGC-1β binds and sustains the activity of SREBP-1c that is a master regulator of lipogenesis (Lin et al., 2005; Ducheix et al., 2016). SREBP-1c overexpression was associated with TC accumulation through stimulating the transcriptions of many lipogenetic genes including FAS, ACC, SCD-1 (Felder et al., 2007). In this study, we found that HSAV significantly elevated the expression of PGC-1β, and decreased the levels of protein and gene involved in SREBP-1c mediated lipigenesis signal pathway, consisting of FAS, ACC and SCD1. It disclosed that HSAV reduced lipid level in T2DM through PGC-1β/SREBP-1c mediated lipogenesis signal pathway.

It has been reported that there is a certain correlation between the activity compounds of aromatic vinegar and its antioxidant activity (Zhang et al., 2019). Aromatic vinegar could inhibit ROS generation in high glucose-induced HepG2 cells, and improve insulin sensitivity and maintains glucose homeostasis (Xia et al., 2021). Given the critical roles of glycolipid metabolism in the pathogenesis of T2DM, we evaluated the regulation effect of HSAV on glycolipid metabolism of T2DM. Despite this, further investigation on the antioxidant activity of HSAV and its clinical application in treating T2DM still need, which will explore more opportunities for the application of HSAV as a clinical adjunctive therapy.

## Conclusion

HSAV showed a potent effect against T2DM, and regulates PGC-1α and PGC-1β mediate glycolipid metabolism pathway, thereby improving glycogen synthesis, reducing gluconeogenesis and lipid synthesis. This study provided a new perspective and evidence for the application of HSAV as diet supplement in T2DM patients and susceptible population.

## Data Availability

The raw data supporting the conclusions of this article will be made available by the authors, without undue reservation.
